# Investigation of altered spontaneous brain activity in patients with bronchial asthma using the percent amplitude of fluctuation method: a resting-state functional MRI study

**DOI:** 10.3389/fnhum.2023.1228541

**Published:** 2023-11-30

**Authors:** Tao Wang, Xin Huang, Li-xue Dai, Kang-min Zhan, Jun Wang

**Affiliations:** ^1^Medical College of Nanchang University, Nanchang, China; ^2^The Second Department of Respiratory Disease, Jiangxi Provincial People’s Hospital, The First Affiliated Hospital of Nanchang Medical College, Nanchang, China; ^3^Department of Ophthalmology, Jiangxi Provincial People’s Hospital, The First Affiliated Hospital of Nanchang Medical College, Nanchang, China

**Keywords:** asthma, PerAF, fMRI, brain activity, cognitive impairment

## Abstract

**Purpose:**

To explore the regions of aberrant spontaneous brain activity in asthma patients and their potential impacts using the Percent amplitude of fluctuation (PerAF) analysis method.

**Patients and methods:**

In this study, a total of 31 bronchial asthma (BA) patients were ultimately included, comprising 17 males and 14 females. Subsequently, 31 healthy control subjects (HCS) were recruited, consisting of 17 males and 14 females, and they were matched with the BA group based on age, sex, and educational status. The PerAF analysis technique was employed to study the differences in spontaneous brain activity between the two groups. The SPM12 toolkit was used to carry out a two sample *t*-test on the collected fMRI data, in order to examine the differences in PerAF values between the asthma patients and the healthy controls. We employed the Montreal Cognitive Assessment (MoCA) scale and the Hamilton Depression Scale (HAMD) to evaluate the cognitive and emotional states of the two groups. Pearson correlation analysis was utilized to ascertain the relationship between changes in the PerAF values within specific brain regions and cognitive as well as emotional conditions.

**Results:**

Compared with the healthy control group, areas of the brain with reduced PerAF in asthma patients included the inferior cerebellum, fusiform gyrus, right inferior orbital frontal gyrus, left middle orbital frontal gyrus, left/right middle frontal gyrus (MFG), dorsal lateral superior frontal gyrus (SFGdl), left superior temporal gyrus (STG), precuneus, right inferior parietal lobule (IPL), and left/right angular gyrus. BA patients exhibit mild cognitive impairments and a propensity for emotional disturbances. Furthermore, the perAF values of the SFGdl region are significantly positively correlated with the results of the MoCA cognitive assessment, while negatively correlated with the HAMD evaluation.

**Conclusion:**

Through the application of PerAF analysis methods, we discovered that several brain regions in asthma patients that control the amplitude of respiration, vision, memory, language, attention, and emotional control display abnormal changes in intrinsic brain activity. This helps characterize the neural mechanisms behind cognitive, sensory, and motor function impairments in asthma patients, providing valuable insights for potential therapeutic targets and disease management strategies.

## 1 Introduction

Bronchial Asthma (BA) is a prevalent illnesses marked by recurrent airway inflammation, increased bronchial responsiveness, and variable airflow restriction (Papi et al., 2018). It is usually associated with air pollution, allergens, microorganisms, and RV virus infections (Miller et al., 2021). The latest nationwide study shows that the prevalence of asthma in China is 4.2%, affecting nearly 45.7 million Chinese adults, a quarter of whom have airflow-limited asthma (Huang et al., 2019). According to a meta-analysis, the incidence of BA among Chinese teenagers (> 14 years old) and adults has grown since 2010 (Jiang et al., 2023). Currently, less than 15% of BA patients are well controlled (Castillo et al., 2017; Ding and Small, 2018). Another meta-analysis on the relationship between BA and cognitive impairment shows that BA patients perform worse in cognitive domains than healthy controls, including spatial memory, visual and tactile motor tasks, delayed recall, explicit memory, language, abstract reasoning, and strategies for responding to changing environmental conditions (Irani et al., 2017). Impaired cognitive function could potentially be a significant determinant of the prognosis for BA. Cognitive deficits can severely impact a patient’s ability to manage their own disease, thereby increasing the risk of acute exacerbations. Research indicates that cognitive impairment is related to the duration of BA symptoms (Rhyou and Nam, 2021).

However, the exact pathogenesis of cognitive impairment in BA patients is not fully understood, with hypoxemia, oxidative stress, and chronic inflammatory responses potentially playing significant roles. The brain is highly sensitive to hypoxia, and chronic hypoxia inevitably leads to neuronal damage, subsequently affecting brain structure and function, and resulting in cognitive impairment (Ren et al., 2021; Rhyou and Nam, 2021). Apart from the direct damage of hypoxia to neurons, the chronic airway inflammation in BA can also induce the activation of neuroglia and promote the expression of inflammatory factors, exacerbating brain neuronal injury (Nair et al., 2022; Rosenkranz et al., 2022a). Kroll et al. (2018) found that the poorer cognitive function in BA patients was associated with decreased metabolites in the hippocampus, such as N-acetylaspartate (NAA) and glutamate (Glu). Rosenkranz et al. (2022a) found in a study that changes occurred in glial fibrillary acidic protein (GFAP) and Neurofilament light (NfL) in the plasma of BA patients, which reflect the extent of neurodegeneration and inflammation in asthma patients. Bian et al. (2018) discovered that the fractional anisotropy (FA) value of the entire brain decreased, indicating abnormal white matter integrity, and these change is related to cognitive impairment and executive dysfunction. Furthermore, the volume of the basal ganglia, amygdala, and hippocampus in asthma patients is reduced, and these structural changes lead to worsening respiratory discomfort and the occurrence of cognitive impairment (Kroll and Ritz, 2023).

Symptoms of BA can trigger specific neural pathways in the brain, leading to changes in endocrine and emotional states, thereby exacerbating symptoms. Research shows that inflammatory mediators produced after inhaling allergens typically stimulate airway nerve endings and activate the insula and anterior cingulate cortex (Busse, 2012). This activation of neural circuits makes the brain more susceptible to abnormal symptom perception, leading to aberrant secretion of catecholamines and dopamine, and further causing an increase in negative emotions and eosinophil proliferation (Rosenkranz and Davidson, 2009; Rosenkranz et al., 2012). Furthermore, sustained negative emotions can lead to the onset of chronic stress. Further research has revealed that chronic stress has been found to result in increased activity in the mid-insular and perigenual anterior cingulate cortex (PACC), which is associated with more severe airway inflammation (Rosenkranz et al., 2016). Additionally, chronic stress can shift the Th1 cell response toward a Th2 pattern, while reducing the responsiveness to corticosteroids and compromising the airway barrier, thereby enhancing allergic airway inflammation (Busse, 2012). Moreover, chronic stress can also activate the amygdala and upregulate the IL-1 pathway, thereby exacerbating the inflammatory environment in the airways (Rosenkranz et al., 2022b). Activation of specific neural circuits such as the ACC and insula often precedes late-stage lung inflammation and airflow obstruction, which can predict late-phase pulmonary responses (Busse, 2012). Asthma sufferers’ altered brain structure and function are attracting increased attention since they are associated with cognitive deficits and symptom progression in these patients.

According to earlier research, patients with BA show changes in the brain’s functional networks, including the executive control network (ECN), default mode network (DMN), salience network (SN), visual network, and sensorimotor network (Li et al., 2018; Wang et al., 2022; Zhang et al., 2022). These alterations in functional networks can explain the abnormal clinical features observed in asthma patients, including visual changes, attention biases, abnormal respiratory sensations, and emotional regulation disorders. However, most of these studies have primarily focused on elucidating the overall dysfunction in the brain regions of BA patients at the network level, overlooking the impact of localized brain region alterations. On the other hand, a more precise identification of localized brain dysfunction in patients can aid in understanding the neural pathways associated with disease progression. In the study of localized functional changes in BA patients’ brains, alterations in regional homogeneity (ReHo) activity have been elucidated, including the temporal and occipital lobes (Huang et al., 2021). In a multimodal rs-fMRI study conducted by Li S. et al. (2020), it was found that BA patients exhibited decreased amplitude of low-frequency fluctuations (ALFF) in the angular gyrus and precuneus, while ALFF in the temporal lobe showed an increase. The study also revealed distinct changes in brain regions and network functionality. These alterations in brain networks and regional functions may potentially serve as underlying biomarkers for cognitive impairment in BA.

However, approaches such as ReHo, amplitude of low-frequency fluctuation (ALFF), and fractional ALFF (FALFF) are easily influenced by physiological high-frequency noises and arbitrary units, making their statistical analysis relatively challenging (Ni et al., 2012). As a new method for observing changes in brain activity, the percentage amplitude fluctuation (PerAF) calculates the percentage of signal fluctuations at each time point relative to the mean, and its results are not affected by magnetic field inhomogeneity. PerAF is not affected by the whole brain average or the amplitude fluctuation of specific voxel frequencies. Furthermore, PerAF has better repeatability and accuracy than ALFF and fALFF (Jia et al., 2020). Moreover, PerAF is more sensitive than ALFF (Yu et al., 2019). Due to the reduction of BOLD signal impact, PerAF surpasses ALFF in terms of scanner reliability (Zhao et al., 2018). PerAF has been utilized in the study of brain disorders and cognitive changes associated with obstructive sleep apnea (Xie et al., 2022).

Although PerAF has great potential in voxel-level analysis, no research has been conducted to explore the spontaneous brain activity of BA patients using PerAF. We speculate that BA patients may exhibit abnormal oscillatory percentage amplitude, and such changes could be associated with cognitive functional alterations. Therefore, we attempted to preliminarily investigate the brain regions with abnormal PerAF in BA patients, aiming to provide more accurate pathological localization for BA patients with comorbid cognitive impairments. Additionally, we further analyzed the cognitive dysfunction associated with these regions to supplement the understanding of BA patients with comorbid cognitive impairments.

## 2 Materials and methods

### 2.1 Clinical data

In this study, a total of 31 bronchial asthma patients were selected from the Jiangxi Provincial People’s Hospital. Additionally, healthy participants were recruited from within Jiangxi Province, and 31 healthy controls were ultimately included. Matching for age, gender, and educational level was ensured among the two groups. Prior to participating in the trial, every subject was given details regarding the study’s aims and gave their informed consent. They underwent MRI with the same imaging parameters.

The chosen asthma cohort fulfills these conditions: (1) Presence of recurrent wheezing, with forced expiratory volume declining over 20% within an hour; (2) Individuals are in a non-acute phase of asthma. (3) No comorbidities with other respiratory system diseases.

The selected HC group fulfills the following conditions: (1) The blood relatives of these subjects show no record of asthma or inherited diseases. (2) We’ve ensured that the BA group is virtually the same in terms of gender, age, and educational attainment.

All participants conformed to the subsequent conditions: (1) MRI scans revealed no clear structural anomalies in the brain parenchyma. (2) There was no prior record of psychiatric conditions, chronic illnesses, benign or malignant growths, or cerebral infarctions. (3) No contraindications for MRI examinations (e.g., pacemakers, cardiac stents). (4) No history of alcoholism, drug addiction, or substance dependence. (5) No claustrophobia and able to tolerate MRI examination. (6) Not pregnant.

### 2.2 Neuropsychological assessment

To ensure the reliability of the measured results, this study arranged for two professional psychologists to conduct the tests. In our study, we employed the Montreal Cognitive Assessment (MoCA) scale as a cognitive function measurement tool. The MoCA demonstrates high sensitivity in detecting mild cognitive impairment (MCI). It assesses visuospatial and executive functions, naming, orientation, abstract thinking, attention, delayed recall, language, and memory (Chen K. L. et al., 2016). The maximum score on the scale is 30 points, with an additional point given if the participant has an education level of ≤ 12 years. Scores between 18 and 26 indicate MCI (Nasreddine et al., 2005). Furthermore, we utilized the 17-item Hamilton Depression Scale (HAMD-17) to assess the emotional status of the two groups of participants (Hamilton, 1960). The test results from this scale will provide a basis for analyzing cognitive and emotional differences among the subjects.

### 2.3 fMRI data acquisition

All magnetic resonance imaging (MRI) scans were conducted at Jiangxi Provincial People’s Hospital. In this study, we used a Trio 3-Tesla MR scanner for the capture of MRI data. Participants were guided to keep their eyes closed and maintain shallow breathing naturally throughout the scanning duration. Functional data was collected using the gradient-recalled echo-planar imaging (GRE-EPI) sequence. A total of 176 structural images and 240 functional images were collected. Standard scanning parameters were applied according to a unified protocol, as shown in Table 1.

**TABLE 1:** The parameters for obtaining fMRI images.

MRI scanning parameters	Structural images	Functional images
Matrix size	256 × 256	64 × 64
Field of view	250 × 250 mm	220 × 220 mm
Echo time	2.26 ms	30 ms
Repetition time	1900 ms	2000 ms
Slice thickness	1.0 mm	4.0 mm
Slice gap	0.5 mm	1.2 mm
Flip angle	9°	90°

### 2.4 Data pre-processing

Initially, participant’s conventional MRI scans were assessed by a pair of physicians to identify and exclude any individuals with noticeable brain structural anomalies. Subsequently, the integrity and quality of MRI data were verified utilizing the MRIcro software suite to remove images with incomplete scan coverage, machine artifacts, and significant metal artifacts. Finally, the BOLD data underwent pre-processing. In this study, we utilized the upgraded rs-fMRI tool (DPARSFA 4.0)^1^ software for data processing. The pre-processing steps included: (1) Convert the format to the neuroimaging Informatics Technology Initiative (NIFTI) format that stores 3 or 4 dimensional images for subsequent processing. (2) The initial 20 s of imagery for each participant were discarded at the commencement of the scan. (3) We employed the interleaved scanning to reconcile discrepancies in the timing of image capture across different layers. (4) Rigid Transformation was utilized to correct for any head movements that exceed 1.5 millimeters or 1.5 degrees in any direction. (5) Functional and structural images are aligned and segmented, with the gray matter component undergoing spatial normalization using the Montreal Neurological Institute (MNI) coordinate system. Resample all voxel units to 3 mm × 3 mm × 3 mm. (6) In order to maintain the inherent resolution, we applied isotropic Gaussian smoothing with a full-width half-height (FWHH) value of 6 mm to the images and removed linear drift to eliminate the interference of noise signals related to the instrument. (7) Confounding covariates including white matter, cerebrospinal fluid, whole brain signal, and Friston 24 head movement parameters were regressed using multivariate regression. (8) The low-frequency component of the BOLD signal was obtained using a band-pass filter (0.01–0.08 Hz). The RESTplus V1.2 software package was used for calculating the whole-brain average PerAF, which involves measuring the PerAF value by assessing the ratio of BOLD signal strength at individual time point against the average BOLD signal strength throughout the complete time series.

### 2.5 Statistical analysis

We utilized SPSS version 27.0 to evaluate the general physiological characteristics, cognition, and psychological scales of both groups. The two-sample *t*-test was used to determine if there were significant differences. In the analysis of the PerAF differences between the two groups, we also used a two-sample *t*-test, considering age, education level, and head movement as covariates. A Gaussian random field (GRF) correction was applied (two-tailed, with a significance threshold at the voxel level of *P* < 0.001 and at the cluster level of *P* < 0.05).

Using the functionality to Extract ROI signals in RESTPlus, brain regions with statistical differences are determined, with peak coordinates as the center and a 6 mm radius to define the ROI. Pearson correlation analysis is performed on the PerAF values of the ROI and clinical cognitive assessments.

## 3 Results

### 3.1 Demographic statistics and clinical scales

In this study, there were no significant distinctions in age, gender, weight, or BMI between the BA and HC groups (*P* > 0.05). The data are presented as mean ± SD. The duration of asthma was 27.15 ± 6.19 years. The HAMD scores for the BA group and the HC group are 8.87 ± 2.99 and 5.94 ± 1.33, respectively, while the MoCA scores for the BA group and the HC group are 22.24 ± 1.88 and 28.92 ± 1.64, respectively. The BA group shows mild cognitive impairment and emotional changes. Please refer to Table 2 for detailed results.

**TABLE 2:** Demographics and clinical measurements by group.

Conditions	BA	HC	t	*P*-value
Male/female	17/14	17/14	N/A	1
Age (years)	52.43 ± 4.3	52.25 ± 4.17	0.167	0.868
Education (years)	12.70 ± 1.33	12.82 ± 1.41	-0.345	0.732
Weight (kg)	64.41 ± 5.14	63.11 ± 4.69	1.040	0.302
BMI (kg/m^2^)	22.76 ± 2.80	21.93 ± 2.83	1.161	0.250
Duration of asthma	27.15 ± 6.19	N/A	N/A	N/A
HAMD score	8.87 ± 2.99	5.94 ± 1.33	4.985	<0.001
MoCA score	22.24 ± 1.88	28.92 ± 1.64	-14.908	<0.001

BA group, bronchial asthma group; HC group, healthy control group; BMI, Body Mass Index; HAMD, Hamilton Depression Scale; MoCA, Montreal Cognitive Assessment; *P* > 0.05.

### 3.2 Differences in PerAF

**FIGURE 1:**
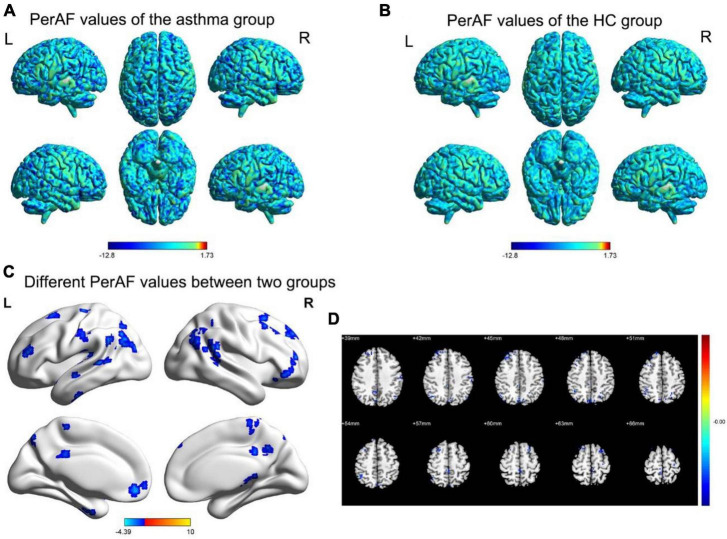
The spatial distribution maps of PerAF values for the BA group **(A)** and HC group **(B)**. **(C,D)** The brain regions with disparate PerAF values are observed in two groups. (*p* < 0.05 for GRF voxel, *p* < 0.05 for clustering, double tailed); **(D)** The corner number of the image shows the coordinates of the relevant MNI space; The yellow and red areas in the legend indicate an increase in the brain area of PerAF; Blue area: Refers to the brain area where PerAF decreases; L: Left; R: Right.

Compared to the healthy control group, the regions with decreased PerAF values include the inferior cerebellum, fusiform gyrus, right orbital inferior frontal gyrus, left orbital middle frontal gyrus, left/right middle frontal gyrus, dorsolateral superior frontal gyrus, left superior temporal gyrus, precuneus, right inferior parietal lobule, left/right angular gyrus (Figure 1 and Table 3).

**TABLE 3:** Brain areas where the BA and HC groups have different perAF values.

Cluster	Regions	Side	Voxels	MNI			*T*-value (Peak intensity)
				**x**	**z**	**y**	
1	Inferior cerebellum	R	13	39	−72	−42	−3.3063
2	Fusiform gyrus	L	12	−33	−9	−27	−3.5388
3	Inferior orbital frontal gyrus	R	14	51	42	−9	−3.2396
4	Middle orbital frontal gyrus	L	16	−3	51	−6	−3.5019
5	Superior temporal gyrus	L	11	−51	−18	0	−3.5193
6	Middle frontal gyrus	R	16	39	51	12	−3.4391
7	Middle frontal gyrus	L	17	−39	42	18	−3.0945
8	Angular gyrus	L	17	−42	−63	33	−4.2443
9	Angular gyrus	R	25	51	−42	24	−4.3858
10	Dorsal lateral superior Frontal gyrus	R	20	30	48	36	−4.3165
11	Precuneus	L	13	−3	−81	45	−3.8876
12	Inferior parietal lobule	R	15	45	−54	51	−3.4457

Gaussian random field theory (*P* < 0.05, cluster > 10 voxels, GRF corrected) was used to multiple comparisons. MNI, Montreal neurological institute.

### 3.3 Correlation analysis

**FIGURE 2:**
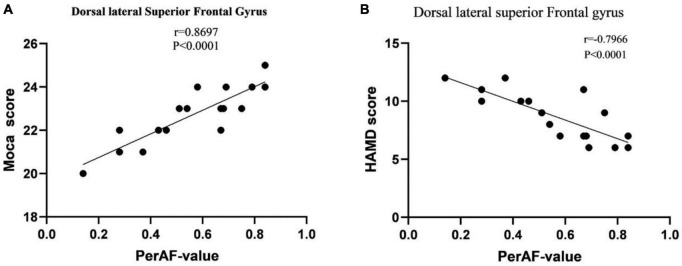
**(A)** Correlation between MoCA scores in BA patients and PerAF signal values. **(B)** Correlation between HAMD scores in BA patients and PerAF signal values. Abbreviations: MoCA, Montreal Cognitive Assessment; HAMD, Hamilton Depression; PerAF, Percentage of Amplitude Fluctuation; BA, Bronchial Asthma.

The results indicate that in BA patients, the perAF value of SFGdl is significantly positively correlated with Moca cognitive assessment (*r* = 0.8697, *p* < 0.0001), and significantly negatively correlated with HAMD assessment (*r* = −0.7966, *p* < 0.0001). The perAF values of other brain regions showed no significant correlation with the neuropsychological assessments (Figure 2).

## 4 Discussion

PerAF has been applied in the study of various neurogenic diseases and is a new, more reliable method compared to ALFF, ReHo, and DC. This method reduces the influence of BOLD signals and units. In this study, we used the PerAF method for the first time to explore the activity changes in several brain regions of asthma patients. The results indicated that compared to HC, the PerAF values in the inferior cerebellum, fusiform gyrus, right orbital inferior frontal gyrus, left orbital middle frontal gyrus, left/right middle frontal gyrus, dorsolateral superior frontal gyrus, left superior temporal gyrus, precuneus, right inferior parietal lobule, left/right angular gyrus in BA patients decreased. However, we did not find brain regions where the PerAF value of BA patients increased. In addition, this study demonstrates a significant positive correlation between the perAF value of the SFGdl and the Moca cognitive assessment, while showing a negative correlation with the HAMD evaluation. There was no significant correlation observed between perAF value in other brain regions and the neurocognitive assessments, suggesting a possible functional compensation in these brain regions. This study found that the BA group showed mild cognitive impairment and emotional changes.

The cerebellum, located in the posterior cranial fossa of the brain, plays an important role in maintaining posture, ensuring balanced gait, and controlling voluntary, precise movements (Stoodley and Schmahmann, 2010). Hypoxia is a common factor causing cerebellar damage (Koeppen, 2018). Li H. et al. (2020) found functional connectivity changes in the cerebellum in people suffering from COPD. The cerebellum is linked to neurotransmitter synthesis because it is an essential region for the production of nitric oxide (NO) (Mayer, 1994; Rodrigo et al., 2004). Furthermore, abnormal NO levels can also impede the development of oligodendrocytes in the cerebellum, which may explain the motor problems observed in patients with persistent chronic hypoxia (Barradas et al., 2016). Obstructive sleep apnea (OSA) is also distinguished by repeated airway blockage and intermittent hypoxia, and these patients usually have reduced cerebellar blood flow (CBF). This may be due to elevated end-tidal carbon dioxide (PECO2) levels, leading to impaired vasodilation and inadequate local perfusion, further worsening respiratory sensation and motor coordination (Yadav et al., 2013). The cerebellum, on the other hand, is associated with communication, executive capability, cognitive capability, and mood regulation in addition to physical balance and movement (Yu et al., 2021). Moreover, the abnormalities in structure and function of the cerebellum have been shown to be associated with the occurrence of negative emotional experience among individuals with Parkinson’s disease (Ma et al., 2018). In our study, the decreased PerAF values in the cerebellum of asthma patients may be associated with respiratory irregularities, decreased executive function, mood regulation disorders, and cognitive deficits. This aligns with our research that the scores for HAMD and MoCA in the asthma group also indicate that they may have mild cognitive impairments and emotional abnormalities. However, the association between these abnormal presentations and the reduced value of cerebellar PerAF needs further investigation for confirmation.

The fusiform gyrus is located at the bottom of the junction between the occipital and temporal lobes (Parise et al., 2014). It is a crucial part of the ventral stream in the visual pathway, primarily involved in tasks such as object naming, memory, face perception, and visual stimulus processing (Koh, 2022; Reindl et al., 2022). Previous reports have suggested that facial processing deficits can lead to increased susceptibility to negative emotional reactions in children with Autism Spectrum Disorder (ASD) (Apicella et al., 2013). A study on anorexia patients revealed that the right fusiform gyrus under positive emotional impact could enhance the BOLD response during facial expression processing (Fonville et al., 2014). Another resting-state fMRI study demonstrated a reduced regional homogeneity (ReHo) in the fusiform gyrus of COPD patients, which was positively correlated with attention (Zeng et al., 2022). Here, we observed a decrease in the PerAF of the fusiform gyrus in patients with asthma, which might affect their social experiences as well as drug recognition.

The Orbitofrontal Cortex (OFC) is located on the ventral surface of the frontal lobe (Fine and Hayden, 2022). It has a paramount function in the interpretation of sensory input, and forming advanced emotional and social intelligence, thereby facilitating decision-making, updating of knowledge, and memory retention (Wallis, 2007). The OFC chiefly influences purposeful actions via the rewarding aspects of reinforcers and correlates with subjective emotional experiences; when OFC is impaired, patients use the inability to perceive changes in physical state as a warning cue to control unwanted behavior, while ceasing to perform in response to incentive or punishment predictions and reducing purposeful actions (Rudebeck and Rich, 2018; Rolls, 2019). Abnormalities within the OFC are observed in a range of mental diseases, such as bipolar condition (Ferguson and Gao, 2018), depression (Lai, 2021), anxiety conditions (Kirlic et al., 2017), obsessive-compulsive syndrome (Nakao et al., 2014), social interaction problems (Evans et al., 2004), and conditions involving drug misuse Stoeckel et al. (2015) demonstrated a correlation between the OFC and the habituation of breathlessness perception in asthma patients. Herigstad et al. (2017) also found that the OFC played a part in the heightened sensitivity of dyspnea in patients with COPD who were participating in breathing therapy. In our study, we observed a decreased perAF value in the OFC of asthma patients, which may affect patients’ treatment compliance, awareness of symptoms, and their ability to perceive their own bodily states, thereby increasing the risk of acute asthma exacerbations.

The Dorsolateral Superior Frontal Gyrus (SFGdl) belongs to the upper region of the dorsolateral prefrontal cortex (dlPFC), and it is highly interconnected with other structures in the PFC (Li et al., 2013). It participates in activities of advanced cognitive functions such as visual processing (Alagapan et al., 2019), mood regulation (Wang et al., 2019) (Johnstone et al., 2007), attention distribution, cognitive control tasks (Hu et al., 2022), somatic balance control (Chen et al., 2023) and the plan for sequential behavior (Mansouri et al., 2007), acting as a key node in the frontoparietal network (FPN). Among people with negative emotional distress, the dorsolateral prefrontal cortex (DLPFC) mediates cognitive control and prioritizes the processing of negative stimuli (Lee et al., 2007). Previous evidence suggests that executive functions improve after transcranial stimulation of the left DLPFC in patients with depression (Boggio et al., 2007). Additionally, the DLPFC probably be associated with lower levels of oxygen saturation (Chen J. et al., 2016). Research also indicates that the superior frontal gyrus may be related to changes in respiratory amplitude (Wu X. et al., 2021). Furthermore, another study found a strong correlation between the volume of the right DLPFC and visual imagery ability, suggesting that abnormalities in this region may lead to visual constructional deficits (Chen J. et al., 2016). Similarly, obsessive-compulsive disorder (OCD) has been closely associated with abnormal activity in the dlPFC (Nakao et al., 2014). We speculate that changes in perAF in this region could not only further disrupt the respiratory rhythm in asthma but also affect patients’ ability to carry out daily activities, including the execution of asthma control plans. It is important to note that functional deficits in this area may lead to diminished inhibition of emotional disturbances in cognitive tasks, as well as compulsive drug-seeking behavior might emerge. Because visual construction defects and working memory impairments could lead to a decrease in the ability of BA patients to identify medications. In our study, we observed a significant correlation between the values of PerAF in the SFGdl and the scores on the neurocognitive assessment scale. Consequently, the PerAF values in the SFGdl could serve as a potential neurobiological biomarker for delineating cognitive and emotional states in individuals with BA.

The Middle Frontal Gyrus (MFG), located between the superior and inferior frontal sulci, plays a vital role in top-down control of visual attention processing. It mediates the reorientation of attention from ongoing endogenous attention processes to new task-relevant external stimuli, thereby acting as a key component of the dorsal and ventral attention networks (Japee et al., 2015). Tafazoli et al. (2013) found that children with attention deficit hyperactivity disorder (ADHD) exhibit MFG deficits, which lead to reduced expression of energy metabolites such as creatine and result in attention impairments. In a study investigating highly myopic patients using voxel-wise degree centrality (DC), a decrease in DC values in the MFG was associated with visual spatial processing and working memory deficits (Hu et al., 2018). Furthermore, the MFG is associated with emergency awareness, which involves recognizing and learning fear stimuli and eliciting conditioned responses, making it crucial for adapting to environmental changes (Knight et al., 2004; Klucken et al., 2009). Additionally, the MFG is involved in language switching through top-down selection inhibition mechanisms (Sierpowska et al., 2018). In our study, we observed a decrease in PerAF values in the MFG. This change in the region could be associated with impaired attention redirection in asthma patients, as well as induce deficits in emergency awareness, reducing their motivation to promptly address symptoms.

The Superior Temporal Gyrus (STG) is situated between the lateral sulcus and the superior temporal sulcus. It primarily handles auditory processing and language comprehension, but also contributes to self-emotional experience and the interpretation of facial cues (Bhaya-Grossman and Chang, 2022). Previous research has shown that cortical volume in the STG has been linked to cognitive abilities including motor skills, attention, and the rate at which information is handled (Achiron et al., 2013). The Wernicke’s area, an important rear region of the STG is the auditory speech center, and abnormalities in this area often result in a lack of meaningful speaking (Javed et al., 2023). In a magnetic resonance imaging study of 21 individuals with social anxiety disorder, it was found that they often have smaller volumes in the STG, and this population tends to receive negative feedback from social interactions (Atmaca et al., 2021). Similarly, another study that conducted brain morphological analysis using MRI in individuals with schizophrenia found similar results (Bandeira et al., 2021). In our study, we discovered that asthma patients had a lower PerAF in the STG. The STG may be implicated in the identification of asthma-related sounds, such as wheezing and rapid breathing. However, asthma patient’s exhibit reduced sensitivity to these auditory stimuli, resulting in a weakened perception of respiratory symptoms. We speculate that long-term incoherent speech expression may also lead to social anxiety in asthma patients, and this is an aspect that caregivers should be aware of in the long-term management of chronic asthma.

The precuneus is anatomically located above the occipital lobe and on the inside of the parietal cortex, is a key area of the Default Mode Network (DMN) (Fletcher et al., 1995). It plays a major role in various highly integrated activities such as visual attention, spatial image imagination, episodic recall extraction, and self-related information processing, which are critical for the development of self-consciousness (Rosemann and Rauschecker, 2022). Furthermore, it assists in simulating non-self-representations from a third-person perspective, that is, the ability to understand relevant behaviors through the intentions of others, which contributes to successful social interactions (Cavanna and Trimble, 2006). Previous studies have indicated that precuneus impairments are present in various working memory and attention-related dysfunction, including Alzheimer’s illnesses (Miners et al., 2016), autism (Kitamura et al., 2021), ADHD (Lukito et al., 2020), schizophrenia (Li et al., 2021), and depression (Szymkowicz et al., 2017). An fMRI study on regional or network-level brain function changes in Social Anxiety Disorder (SAD) revealed that the precuneus was associated with SAD-related abnormal dread and evasion, as well as the formation of negative emotions (Yuan et al., 2018). Zhang et al. found that the precuneus may be involved in rating respiratory difficulties in COPD patients (Herigstad et al., 2015). We hypothesize that this is primarily due to the influence of the precuneus on internal self-representations of respiratory difficulties. Several previous studies investigating brain network alterations in asthma have also found abnormal activity in the precuneus, but these discussions have mostly been at the network level, which may not precisely elucidate the impact of regional functional changes on symptoms (Li S. et al., 2020; Zhu et al., 2023). Changes in the precuneus may affect the ability of patients to attend to and process visual spatial cues related to asthma management, such as remembering how to use medication or recognizing the need for self-medication, while also affecting normal social interactions. The impact of the precuneus on BA should be emphasized in future studies. Notably, we observed a decrease in PerAF in the inferior parietal lobule (IPL) in our study. Located below the intraparietal sulcus, the IPL is involved in somatic perception, reading comprehension, computation, knowledge extraction, cognitive control, and movement orientation. It is also one of the main brain regions in the DMN (Wu Y. J. et al., 2021). The IPL appears to be pivotal in reacting to new and noteworthy stimuli, which forms the basis for reconfiguring task goals related to prominent objects (Singh-Curry and Husain, 2009). The IPL-mediated endogenous processes have high predictive value for the accuracy and utilization of cue information in spatial attentional control (Sapir et al., 2005). Furthermore, second language learning relies on the functional plasticity of the IPL itself (Barbeau et al., 2017). IPL damage can cause significant cognitive problems including as agnosia, memory decline, and aphasia (Ardila, 2020; Humphreys et al., 2021; Sakurai et al., 2021). In addition, damage to the Inferior Parietal Lobule (IPL) can also cause functional disability of limbs (Singh-Curry and Husain, 2009). Liu et al. (2014) found a reduction in the right IPL activity in Alzheimer’s disease (AD) patients, which correlated positively with impaired attention. Similarly, Yu et al. (2021) observed a decrease in the ALFF value of the IPL in COPD patients, suggesting that the IPL region could serve as one of the diagnostic indicators. However, intriguingly, prior fMRI research on asthma discovered enhanced functional activity in the IPL region (Li et al., 2018; Wu Y. J. et al., 2021). This variation could be attributable to different study parameters and the inclusion of patients at different stages of disease progression. During the initial phases of asthma, cognitive deficits are relatively mild, and there may be compensatory mechanisms at play. Despite perAF has high reproducibility and is less affected by sampling bias, the differences in the related results still need to be further explored. In light of this, we propose that the observed decrease in perAF in the BA patients may indicate abnormalities in their attention control, somatic perception, movement, and learning abilities, thereby affecting their overall management of the disease. We consider the IPL to be a crucial region in the neural regulation of BA patients.

As a key region of the Default Mode Network (DMN), functional abnormalities in the Inferior Parietal Lobule (IPL) and the precuneus could lead to changes in the overall network cooperation. The DMN exhibits persistent activity when the brain is at rest, playing a significant role in maintaining consciousness and emotional cognition (Gonen et al., 2020). Unfortunately, no one has yet analyzed how asthma affects the DMN. Numerous studies to date have observed changes in the functional connectivity of the DMN in patients with Bronchial Asthma (BA), and preliminarily explored the connection between these changes and emotional and cognitive impairments (Li et al., 2018; Li S. et al., 2020). We believe that besides the inhibition of DMN activation by hypoxia itself, other network changes such as interruptions in the switching process between the Default Mode Network (DMN) and the Frontoparietal Network (FPN) mediated by the Salience Network (SN) (Chiong et al., 2013). Moreover, previous studies have shown a significant correlation between sleep quality and DMN function, and patients with BA often have a certain degree of sleep disorders (Wang et al., 2015; Reiter et al., 2022). These changes may be important factors causing a decrease in DMN activity due to asthma. Based on this, further research is needed in the future to clarify the mechanism of asthma’s impact on DMN, providing direction for potential targets to improve abnormalities in the DMN.

### 4.1 Insufficient at present

This study requires some improvements. Firstly, it is necessary to refine the categorization of asthma subjects and expand the number of participants to improve the accuracy of experimental results. Secondly, our cross-sectional study cannot reflect the progression of asthma. Subsequent research should focus on longitudinal evaluations to investigate the relevant brain structural changes in asthma patients before and after treatment. Additionally, it is critical to continue developing and validating classification and diagnostic models to provide theoretical evidence for the effectiveness of clinical treatment plans. Furthermore, In our study, we neglected to analyze the relationship between PerAF values in asthma patients and clinical symptoms, such as respiratory capacity, breathlessness scores, and variations in neurocognitive domains. To further investigate particular biological indicators of asthma, future studies can incorporate multimodal neuroimaging techniques.

## 5 Conclusion

In conclusion, our study, utilizing the PerAF analysis method, demonstrates that asthma patients exhibit significant intrinsic brain activity alterations in multiple cortical regions involved in respiratory control, vision, memory, language, attention, and emotion control when compared to the healthy control group. These findings contribute to characterizing the neurobiological mechanisms underlying cognitive, sensory, and motor impairments in asthma patients, and provide valuable insights for potential therapeutic targets and disease management strategies.

## Data availability statement

The original contributions presented in this study are included in this article/supplementary material, further inquiries can be directed to the corresponding authors.

## Ethics statement

The studies involving humans were approved by Ethics Review Committee of Jiangxi Provincial People’s Hospital. The studies were conducted in accordance with the local legislation and institutional requirements. The participants provided their written informed consent to participate in this study. Written informed consent was obtained from the individual(s) for the publication of any potentially identifiable images or data included in this article.

## Author contributions

TW, XH, and JW mentioned provided a significant, direct, and intellectual contribution to the work, including but not limited to research design, execution, data gathering, analysis, interpretation, writing, modification, review, and publication approval. All authors contributed to the article and approved the submitted version.

## Footnotes

1
http://rfmri.org/DPARSF

## References

[B1] Achiron A. Chapman J. Tal S. Bercovich E. Gil H. Achiron A. (2013). Superior temporal gyrus thickness correlates with cognitive performance in multiple sclerosis. *Brain Struct. Funct.* 218 943–950. 22790785 10.1007/s00429-012-0440-3

[B2] Alagapan S. Lustenberger C. Hadar E. Shin H. W. Fröhlich F. (2019). Low-frequency direct cortical stimulation of left superior frontal gyrus enhances working memory performance. *NeuroImage* 184 697–706. 30268847 10.1016/j.neuroimage.2018.09.064 PMC6240347

[B3] Apicella F. Sicca F. Federico R. R. Campatelli G. Muratori F. (2013). Fusiform gyrus responses to neutral and emotional faces in children with autism spectrum disorders: a high density ERP study. *Behav. Brain Res.* 251 155–162. 23124137 10.1016/j.bbr.2012.10.040

[B4] Ardila A. (2020). Gerstmann Syndrome. *Curr. Neurol. Neurosci. Rep.* 20:48. 10.1007/s11910-020-01069-9 32852667

[B5] Atmaca M. Koc M. Aslan S. Mermi O. Korkmaz S. Gurok M. G. (2021). Superior temporal gyrus volumes in patients with social anxiety disorder. *Prim. Care Comp. CNS Disord.* 23:20m02815. 10.4088/PCC.20m02815 34449986

[B6] Bandeira I. D. Barouh J. L. Bandeira I. D. Quarantini L. (2021). Analysis of the superior temporal gyrus as a possible biomarker in schizophrenia using voxel-based morphometry of the brain magnetic resonance imaging: a comprehensive review. *CNS Spectrums* 26 319–325. 31918770 10.1017/S1092852919001810

[B7] Barbeau E. B. Chai X. J. Chen J. K. Soles J. Berken J. Baum S. (2017). The role of the left inferior parietal lobule in second language learning: An intensive language training fMRI study. *Neuropsychologia* 98 169–176. 27725166 10.1016/j.neuropsychologia.2016.10.003

[B8] Barradas P. C. Savignon T. MANHãES A. C. TENóRIO F. Da C. A. Cunha-Rodrigues M. C. (2016). Prenatal Systemic Hypoxia-Ischemia and Oligodendroglia Loss in Cerebellum. *Adv. Exp. Med. Biol.* 949 333–345. 27714697 10.1007/978-3-319-40764-7_16

[B9] Bhaya-Grossman I. Chang E. F. (2022). Speech Computations of the Human Superior Temporal Gyrus. *Annu. Rev. Psychol.* 73 79–102. 34672685 10.1146/annurev-psych-022321-035256 PMC9447996

[B10] Bian R. Zhang Y. Yang Y. Yin Y. Zhao X. Chen H. (2018). White matter integrity disruptions correlate with cognitive impairments in asthma. *J. Magn. Reson. Imaging* 10.1002/jmri.25946 29356252

[B11] Boggio P. S. Bermpohl F. Vergara A. O. Muniz A. L. Nahas F. H. Leme P. B. (2007). Go-no-go task performance improvement after anodal transcranial DC stimulation of the left dorsolateral prefrontal cortex in major depression. *J. Affect. Disord.* 101 91–98. 17166593 10.1016/j.jad.2006.10.026

[B12] Busse W. W. (2012). The brain and asthma: what are the linkages? *Chem. Immunol. Allergy* 98 14–31. 22767055 10.1159/000336495

[B13] Castillo J. R. Peters S. P. Busse W. W. (2017). Asthma Exacerbations: Pathogenesis, Prevention, and Treatment. *J. Allergy Clin. Immunol. Pract.* 5 918–927. 28689842 10.1016/j.jaip.2017.05.001 PMC5950727

[B14] Cavanna A. E. Trimble M. R. (2006). The precuneus: a review of its functional anatomy and behavioural correlates. *Brain* 129 564–583. 16399806 10.1093/brain/awl004

[B15] Chen J. Lin I. T. Zhang H. Lin J. Zheng S. Fan M. (2016). Reduced cortical thickness, surface area in patients with chronic obstructive pulmonary disease: a surface-based morphometry and neuropsychological study. *Brain Imaging Behav.* 10 464–476. 25986304 10.1007/s11682-015-9403-7

[B16] Chen K. L. Xu Y. Chu A. Q. Ding D. Liang X. N. Nasreddine Z. S. (2016). Validation of the Chinese Version of Montreal Cognitive Assessment Basic for Screening Mild Cognitive Impairment. *J. Am. Geriatr. Soc.* 64 e285–e290. 27996103 10.1111/jgs.14530

[B17] Chen Y. Guo Z. Wang Y. Yin H. Zhang S. Liu W. (2023). Structural and functional differences of the thalamus between drug-naïve Parkinson’s disease motor subtypes. *Front. Neurol.* 14:1102927. 10.3389/fneur.2023.1102927 37265464 PMC10229767

[B18] Chiong W. Wilson S. M. D’Esposito M. Kayser A. S. Grossman S. N. Poorzand P. (2013). The salience network causally influences default mode network activity during moral reasoning. *Brain* 136 1929–1941. 23576128 10.1093/brain/awt066 PMC3673466

[B19] Ding B. Small M. (2018). Disease burden of mild asthma in China. *Respirology* 23 369–377. 29052915 10.1111/resp.13189

[B20] Evans D. W. Lewis M. D. Iobst E. (2004). The role of the orbitofrontal cortex in normally developing compulsive-like behaviors and obsessive-compulsive disorder. *Brain Cogn.* 55 220–234. 15134855 10.1016/S0278-2626(03)00274-4

[B21] Ferguson B. R. Gao W. J. P. V. (2018). Interneurons: Critical regulators of E/I balance for prefrontal cortex-dependent behavior and psychiatric disorders. *Front. Neural Circuits* 12:37. 10.3389/fncir.2018.00037 29867371 PMC5964203

[B22] Fine J. M. Hayden B. Y. (2022). The whole prefrontal cortex is premotor cortex. *Philos. Trans. R. Soc. London Ser. B Biol. Sci.* 377:20200524. 10.1098/rstb.2020.0524 PMC8710885 34957853

[B23] Fletcher P. C. Frith C. D. Baker S. C. Shallice T. Frackowiak R. S. Dolan R. J. (1995). The mind’s eye–precuneus activation in memory-related imagery. *NeuroImage* 2 195–200. 9343602 10.1006/nimg.1995.1025

[B24] Fonville L. Giampietro V. Surguladze S. Williams S. Tchanturia K. (2014). Increased BOLD signal in the fusiform gyrus during implicit emotion processing in anorexia nervosa. *NeuroImage Clin.* 4 266–273. 24501698 10.1016/j.nicl.2013.12.002 PMC3913832

[B25] Gonen O. M. Kwan P. O’Brien T. J. Lui E. Desmond P. M. (2020). Resting-state functional MRI of the default mode network in epilepsy. *Epilepsy Behav.* 111:107308. 10.1016/j.yebeh.2020.107308 32698105

[B26] Hamilton M. (1960). A rating scale for depression. *J. Neurol. Neurosurg. Psychiatry* 23 56–62. 14399272 10.1136/jnnp.23.1.56 PMC495331

[B27] Herigstad M. Faull O. K. Hayen A. Evans E. Hardinge F. M. Wiech K. (2017). Treating breathlessness via the brain: changes in brain activity over a course of pulmonary rehabilitation. *Eur. Respir. J.* 50: 1701029. 10.1183/13993003.01029-2017 PMC5678895 28899937

[B28] Herigstad M. Hayen A. Evans E. Hardinge F. M. Davies R. J. Wiech K. (2015). Dyspnea-related cues engage the prefrontal cortex: evidence from functional brain imaging in COPD. *Chest* 148 953–961. 26134891 10.1378/chest.15-0416 PMC4594628

[B29] Hu J. J. Jiang N. Chen J. Ying P. Kang M. Xu S. H. (2022). Altered regional homogeneity in patients with congenital blindness: a resting-state functional magnetic resonance imaging study. *Front. Psychiatry* 13:925412. 10.3389/fpsyt.2022.925412 35815017 PMC9256957

[B30] Hu Y. X. He J. R. Yang B. Huang X. Li Y. P. Zhou F. Q. (2018). Abnormal resting-state functional network centrality in patients with high myopia: evidence from a voxel-wise degree centrality analysis. *Int. J. Ophthalmol.* 11 1814–1820. 30450313 10.18240/ijo.2018.11.13 PMC6232325

[B31] Huang H. Li S. Y. Shi L. Huang X. Wang J. (2021). Altered spontaneous brain activity in patients with asthma: a resting-state functional MRI study using regional homogeneity analysis. *Neuroreport* 32 1403–1407. 34743166 10.1097/WNR.0000000000001736

[B32] Huang K. Yang T. Xu J. Yang L. Zhao J. Zhang X. (2019). Prevalence, risk factors, and management of asthma in China: a national cross-sectional study. *Lancet* 394 407–418. 31230828 10.1016/S0140-6736(19)31147-X

[B33] Humphreys G. F. Lambon R. M. Simons J. S. A. (2021). Unifying account of angular gyrus contributions to episodic and semantic cognition. *Trends Neurosci.* 44 452–463. 33612312 10.1016/j.tins.2021.01.006

[B34] Irani F. Barbone J. M. Beausoleil J. Gerald L. (2017). Is asthma associated with cognitive impairments? A meta-analytic review. *J. Clin. Exp. Neuropsychology* 39 965–978. 10.1080/13803395.2017.1288802 28325118

[B35] Japee S. Holiday K. Satyshur M. D. Mukai I. Ungerleider L. G. (2015). A role of right middle frontal gyrus in reorienting of attention: a case study. *Front. Syst. Neurosci.* 9:23. 10.3389/fnsys.2015.00023 25784862 PMC4347607

[B36] Javed K. Reddy V. J. Wroten M. (2023). *Neuroanatomy, Wernicke Area [M].* Treasure Island (FL): StatPearls. 30422593

[B37] Jia X. Z. Sun J. W. Ji G. J. Liao W. Lv Y. T. Wang J. (2020). Percent amplitude of fluctuation: A simple measure for resting-state fMRI signal at single voxel level. *PLoS One* 15 e0227021. 10.1371/journal.pone.0227021 31914167 PMC6948733

[B38] Jiang Y. Yue Q. An R. Tie Z. Liu Y. Yu L. (2023). A systematic review and meta-analysis of the prevalence and epidemiology of asthma in people over 14 years of age in China. *J. Asthma* 60 1960–1966. 37074261 10.1080/02770903.2023.2203755

[B39] Johnstone T. Van R. C. Urry H. L. Kalin N. H. Davidson R. J. (2007). Failure to regulate: counterproductive recruitment of top-down prefrontal-subcortical circuitry in major depression. *J. Neurosci.* 27 8877–8884. 17699669 10.1523/JNEUROSCI.2063-07.2007 PMC6672169

[B40] Kirlic N. Aupperle R. L. Misaki M. Kuplicki R. Alvarez R. P. (2017). Recruitment of orbitofrontal cortex during unpredictable threat among adults at risk for affective disorders. *Brain Behav.* 7 e00757. 10.1002/brb3.757 PMC5561318 28828218

[B41] Kitamura S. Makinodan M. Matsuoka K. Takahashi M. Yoshikawa H. Ishida R. (2021). Association of adverse childhood experiences and precuneus volume with intrusive reexperiencing in autism spectrum disorder. *Autism Res.* 14 1886–1895. 34185397 10.1002/aur.2558

[B42] Klucken T. Tabbert K. Schweckendiek J. Merz C. J. Kagerer S. Vaitl D. (2009). Contingency learning in human fear conditioning involves the ventral striatum. *Hum. Brain Mapp.* 30 3636–3644. 19384886 10.1002/hbm.20791 PMC6871066

[B43] Knight D. C. Cheng D. T. Smith C. N. Stein E. A. Helmstetter F. J. (2004). Neural substrates mediating human delay and trace fear conditioning. *J. Neurosci.* 24 218–228. 14715954 10.1523/JNEUROSCI.0433-03.2004 PMC6729570

[B44] Koeppen A. H. (2018). The neuropathology of the adult cerebellum. *Handb. Clin. Neurol.* 154 129–149. 29903436 10.1016/B978-0-444-63956-1.00008-4 PMC6279249

[B45] Koh Y. H. (2022). Right Fusiform Gyrus Infarct with Acute Prosopagnosia. *Acta Neurol. Taiwan.* 31 186–187. 35470413

[B46] Kroll J. L. Ritz T. (2023). Asthma, the central nervous system, and neurocognition: Current findings, potential mechanisms, and treatment implications. *Neurosci. Biobehav. Revi.* 146:105063. 10.1016/j.neubiorev.2023.105063 36708797

[B47] Kroll J. L. Steele A. M. Pinkham A. E. Choi C. Khan D. A. Patel S. V. (2018). Hippocampal metabolites in asthma and their implications for cognitive function. *NeuroImage Clin.* 19 213–221. 30035015 10.1016/j.nicl.2018.04.012 PMC6051470

[B48] Lai C. H. (2021). Fronto-limbic neuroimaging biomarkers for diagnosis and prediction of treatment responses in major depressive disorder. *Prog. Neuro-psychopharmacol. Biol. Psychiatry* 107:110234. 10.1016/j.pnpbp.2020.110234 33370569

[B49] Lee B. T. Seong W. C. Hyung S. K. Lee B. C. Choi I. G. Lyoo I. K. (2007). The neural substrates of affective processing toward positive and negative affective pictures in patients with major depressive disorder. *Prog. Neuro-psychopharmacol. Biol. Psychiatry* 31 1487–1492. 10.1016/j.pnpbp.2007.06.030 17688985

[B50] Li H. Xin H. Yu J. Yu H. Zhang J. Wang W. (2020). Abnormal intrinsic functional hubs and connectivity in stable patients with COPD: a resting-state MRI study. *Brain Imaging Behav.* 14 573–585. 31187474 10.1007/s11682-019-00130-7 PMC7160072

[B51] Li P. Zhou M. Yan W. Du J. Lu S. Xie S. (2021). Altered resting-state functional connectivity of the right precuneus and cognition between depressed and non-depressed schizophrenia. *Psychiatry Res. Neuroimaging* 317:111387. 10.1016/j.pscychresns.2021.111387 34509807

[B52] Li Q. G. Zhou F. Q. Huang X. Zhou X. Liu C. Zhang T. (2018). Alterations of resting-state functional network centrality in patients with asthma: evidence from a voxel-wise degree centrality analysis. *Neuroreport* 29 1151–1156. 29975256 10.1097/WNR.0000000000001087

[B53] Li S. Lv P. He M. Zhang W. Liu J. Gong Y. (2020). Cerebral regional and network characteristics in asthma patients: a resting-state fMRI study. *Front. Med.* 14 792–801. 10.1007/s11684-020-0745-1 32270434

[B54] Li W. Qin W. Liu H. Fan L. Wang J. Jiang T. (2013). Subregions of the human superior frontal gyrus and their connections. *NeuroImage* 78 46–58. 23587692 10.1016/j.neuroimage.2013.04.011

[B55] Liu X. Wang S. Zhang X. Wang Z. Tian X. He Y. (2014). Abnormal amplitude of low-frequency fluctuations of intrinsic brain activity in Alzheimer’s disease. *J. Alzheimer Dis.* 40 387–397. 10.3233/JAD-131322 24473186

[B56] Lukito S. Norman L. Carlisi C. Radua J. Hart H. Simonoff E. (2020). Comparative meta-analyses of brain structural and functional abnormalities during cognitive control in attention-deficit/hyperactivity disorder and autism spectrum disorder. *Psychol. Med.* 50 894–919. 32216846 10.1017/S0033291720000574 PMC7212063

[B57] Ma X. Su W. Li S. Li C. Wang R. Chen M. (2018). Cerebellar atrophy in different subtypes of Parkinson’s disease. *J. Neurol. Sci.* 392 105–112. 30036781 10.1016/j.jns.2018.06.027

[B58] Mansouri F. A. Buckley M. J. Tanaka K. (2007). Mnemonic function of the dorsolateral prefrontal cortex in conflict-induced behavioral adjustment. *Science* 318 987–990. 17962523 10.1126/science.1146384

[B59] Mayer B. (1994). Regulation of nitric oxide synthase and soluble guanylyl cyclase. *Cell Biochem. Funct.* 12 167–177. 7525099 10.1002/cbf.290120304

[B60] Miller R. L. Grayson M. H. Strothman K. (2021). Advances in asthma: New understandings of asthma’s natural history, risk factors, underlying mechanisms, and clinical management. *J. Allergy Clin. Immunol.* 148 1430–1441. 34655640 10.1016/j.jaci.2021.10.001

[B61] Miners J. S. Palmer J. C. Love S. (2016). Pathophysiology of Hypoperfusion of the Precuneus in Early Alzheimer’s Disease. *Brain Pathol.* 26 533–541. 26452729 10.1111/bpa.12331 PMC4982069

[B62] Nair A. K. Van H. C. Bendlin B. B. Zetterberg H. Blennow K. Wild N. (2022). Asthma amplifies dementia risk: Evidence from CSF biomarkers and cognitive decline. *Alzheimers Dement.* 8 e12315. 10.1002/trc2.12315 PMC9270636 35846157

[B63] Nakao T. Okada K. Kanba S. (2014). Neurobiological model of obsessive-compulsive disorder: evidence from recent neuropsychological and neuroimaging findings. *Psychiatry Clin. Neurosci.* 68 587–605. 24762196 10.1111/pcn.12195

[B64] Nasreddine Z. S. Phillips N. A. BéDIRIAN V. Charbonneau S. Whitehead V. Collin I. (2005). The Montreal Cognitive Assessment, MoCA: a brief screening tool for mild cognitive impairment. *J. Am. Geriatr. Soc.* 53 695–699. 15817019 10.1111/j.1532-5415.2005.53221.x

[B65] Ni L. Qi R. Zhang L. J. Zhong J. Zheng G. Zhang Z. (2012). Altered regional homogeneity in the development of minimal hepatic encephalopathy: a resting-state functional MRI study. *PLoS One* 7 e42016. 10.1371/journal.pone.0042016 22848692 PMC3404989

[B66] Papi A. Brightling C. Pedersen S. E. Reddel H. K. (2018). Asthma. *Lancet* 391 783–800. 29273246 10.1016/S0140-6736(17)33311-1

[B67] Parise M. Kubo T. T. Doring T. M. Tukamoto G. Vincent M. Gasparetto E. L. (2014). Cuneus and fusiform cortices thickness is reduced in trigeminal neuralgia. *J. Headache Pain* 15:17. 10.1186/1129-2377-15-17 PMC3997919 24661349

[B68] Reindl C. Allgäuer A. L. Kleiser B. A. Onugoren M. D. Lang J. D. Welte T. M. (2022). Resection of dominant fusiform gyrus is associated with decline of naming function when temporal lobe epilepsy manifests after the age of five: A voxel-based lesion-symptom mapping study. *NeuroImage Clin.* 35:103129. 10.1016/j.nicl.2022.103129 PMC9421498 36002957

[B69] Reiter J. Ramagopal M. Gileles-Hillel A. Forno E. (2022). Sleep disorders in children with asthma. *Pediatric Pulmonol.* 57 1851–1859. 10.1002/ppul.25264 PMC8408281 33647191

[B70] Ren M. Feng M. Long Z. Ma J. Peng X. He G. (2021). Allergic Asthma-Induced Cognitive Impairment is Alleviated by Dexamethasone. *Front. Pharmacol.* 12:680815. 10.3389/fphar.2021.680815 34248632 PMC8261293

[B71] Rhyou H. I. Nam Y. H. (2021). Association between cognitive function and asthma in adults. *Ann. Allergy Asthma Immunol.* 126 69–74. 32858237 10.1016/j.anai.2020.08.022

[B72] Rodrigo J. Fernández A. P. Alonso D. Serrano J. Fernández-Vizarra P. Martínez-Murillo R. (2004). Nitric oxide in the rat cerebellum after hypoxia/ischemia. *Cerebellum* 3 194–203. 15686097 10.1080/14734220410017941

[B73] Rolls E. T. (2019). The orbitofrontal cortex and emotion in health and disease, including depression. *Neuropsychologia* 128 14–43. 28951164 10.1016/j.neuropsychologia.2017.09.021

[B74] Rosemann S. Rauschecker J. P. (2022). Neuroanatomical alterations in middle frontal gyrus and the precuneus related to tinnitus and tinnitus distress. *Hear. Res.* 424:108595. 10.1016/j.heares.2022.108595 35963187

[B75] Rosenkranz M. A. Busse W. W. Sheridan J. F. Crisafi G. M. Davidson R. J. (2012). Are there neurophenotypes for asthma? Functional brain imaging of the interaction between emotion and inflammation in asthma. *PLoS One* 7 e40921. 10.1371/journal.pone.0040921 22870208 PMC3411610

[B76] Rosenkranz M. A. Davidson R. J. (2009). Affective neural circuitry and mind-body influences in asthma. *NeuroImage* 47 972–980. 19465136 10.1016/j.neuroimage.2009.05.042 PMC2748325

[B77] Rosenkranz M. A. Dean D. C. 3RD Bendlin B. B. Jarjour N. N. Esnault S. Zetterberg H. (2022a). Neuroimaging and biomarker evidence of neurodegeneration in asthma. *J. Allergy Clin. Immunol.* 149 589–598. 34536414 10.1016/j.jaci.2021.09.010 PMC8821112

[B78] Rosenkranz M. A. Esnault S. Christian B. T. Crisafi G. Gresham L. K. Higgins A. T. (2016). Mind-body interactions in the regulation of airway inflammation in asthma: A PET study of acute and chronic stress. *Brain Behav. Immun.* 58 18–30. 27039241 10.1016/j.bbi.2016.03.024 PMC5045317

[B79] Rosenkranz M. A. Esnault S. Gresham L. Davidson R. J. Christian B. T. Jarjour N. N. (2022b). Role of amygdala in stress-induced upregulation of airway IL-1 signaling in asthma. *Biol. Psychol.* 167:108226. 10.1016/j.biopsycho.2021.108226 PMC9426565 34800561

[B80] Rudebeck P. H. Rich E. L. (2018). Orbitofrontal cortex. *Curr. Biol.* 28 R1083–R1088. 30253144 10.1016/j.cub.2018.07.018 PMC9253859

[B81] Sakurai T. Hirano S. Abe M. Uji Y. Shimizu K. Suzuki M. (2021). Dysfunction of the left angular gyrus may be associated with writing errors in ALS. *Amyotrop. Lateral Scler. Frontotemp. Degener.* 22 267–275. 10.1080/21678421.2020.1861021 33331163

[B82] Sapir A. D’Avossa G. Mcavoy M. Shulman G. L. Corbetta M. (2005). Brain signals for spatial attention predict performance in a motion discrimination task. *Proc. Natl. Acad. Sci. U. S. A.* 102 17810–17815. 16306268 10.1073/pnas.0504678102 PMC1308888

[B83] Sierpowska J. Fernandez-Coello A. Gomez-Andres A. Camins À Castañer S. Juncadella M. (2018). Involvement of the middle frontal gyrus in language switching as revealed by electrical stimulation mapping and functional magnetic resonance imaging in bilingual brain tumor patients. *Cortex* 99 78–92. 29197227 10.1016/j.cortex.2017.10.017

[B84] Singh-Curry V. Husain M. (2009). The functional role of the inferior parietal lobe in the dorsal and ventral stream dichotomy. *Neuropsychologia* 47 1434–1448. 19138694 10.1016/j.neuropsychologia.2008.11.033 PMC2697316

[B85] Stoeckel M. C. Esser R. W. Gamer M. Büchel C. Von L. A. (2015). Brain mechanisms of short-term habituation and sensitization toward dyspnea. *Front. Psychol.* 6:748. 10.3389/fpsyg.2015.00748 26082746 PMC4451234

[B86] Stoodley C. J. Schmahmann J. D. (2010). Evidence for topographic organization in the cerebellum of motor control versus cognitive and affective processing. *Cortex* 46 831–844. 20152963 10.1016/j.cortex.2009.11.008 PMC2873095

[B87] Szymkowicz S. M. Dotson V. M. Mclaren M. E. De W. L. O’Shea D. M. Talty F. T. (2017). Precuneus abnormalities in middle-aged to older adults with depressive symptoms: An analysis of BDI-II symptom dimensions. *Psychiatry Res. Neuroimaging* 268 9–14. 28837829 10.1016/j.pscychresns.2017.08.002 PMC5593781

[B88] Tafazoli S. O’Neill J. Bejjani A. Ly R. Salamon N. Mccracken J. T. (2013). 1H MRSI of middle frontal gyrus in pediatric ADHD. *J. Psychiatr. Res.* 47 505–512. 23273650 10.1016/j.jpsychires.2012.11.011 PMC3609653

[B89] Wallis J. D. (2007). Orbitofrontal cortex and its contribution to decision-making. *Annu. Rev. Neurosci.* 30 31–56. 17417936 10.1146/annurev.neuro.30.051606.094334

[B90] Wang S. Zhao Y. Zhang L. Wang X. Wang X. Cheng B. (2019). Stress and the brain: Perceived stress mediates the impact of the superior frontal gyrus spontaneous activity on depressive symptoms in late adolescence. *Hum. Brain Mapp.* 40 4982–4993. 31397949 10.1002/hbm.24752 PMC6865488

[B91] Wang T. Huang X. Wang J. (2022). Asthma’s effect on brain connectivity and cognitive decline. *Front. Neurol.* 13:1065942. 10.3389/fneur.2022.1065942 36818725 PMC9936195

[B92] Wang Y. Liu H. Hitchman G. Lei X. (2015). Module number of default mode network: inter-subject variability and effects of sleep deprivation. *Brain Res.* 1596 69–78. 25446443 10.1016/j.brainres.2014.11.007

[B93] Wu X. Zhang Y. Luo W. T. Mai R. R. Hou X. Y. Xia Z. Q. (2021). Brain functional mechanisms determining the efficacy of transcutaneous auricular vagus nerve stimulation in primary Insomnia. *Front. Neurosci.* 15:609640. 10.3389/fnins.2021.609640 33776631 PMC7994340

[B94] Wu Y. J. Rao J. Huang X. Wu N. Shi L. Huang H. (2021). Impaired Interhemispheric Synchrony in Bronchial Asthma. *Int. J. Gener. Med.* 14 10315–10325. 10.2147/IJGM.S343269 PMC8713883 34992446

[B95] Xie W. Shu Y. Liu X. Li K. Li P. Kong L. (2022). Abnormal Spontaneous Brain Activity and Cognitive Impairment in Obstructive Sleep Apnea. *Nat. Sci. Sleep* 14 1575–1587. 36090000 10.2147/NSS.S376638 PMC9462436

[B96] Yadav S. K. Kumar R. Macey P. M. Richardson H. L. Wang D. J. Woo M. A. (2013). Regional cerebral blood flow alterations in obstructive sleep apnea. *Neurosci. Lett.* 555 159–164. 24076138 10.1016/j.neulet.2013.09.033 PMC3891908

[B97] Yu J. Wang W. Peng D. Luo J. Xin H. Yu H. (2021). Intrinsic low-frequency oscillation changes in multiple-frequency bands in stable patients with chronic obstructive pulmonary disease. *Brain Imaging Behav.* 15 1922–1933. 32880076 10.1007/s11682-020-00385-5

[B98] Yu Y. Li Z. Lin Y. Yu J. Peng G. Zhang K. (2019). Depression affects intrinsic brain activity in patients with mild cognitive impairment. *Front. Neurosci.* 13:1333. 10.3389/fnins.2019.01333 31920500 PMC6928005

[B99] Yuan C. Zhu H. Ren Z. Yuan M. Gao M. Zhang Y. (2018). Precuneus-related regional and network functional deficits in social anxiety disorder: A resting-state functional MRI study. *Compreh. Psychiatry* 82 22–29. 10.1016/j.comppsych.2017.12.002 29367059

[B100] Zeng F. Hong W. Zha R. Li Y. Jin C. Liu Y. (2022). Smoking related attention alteration in chronic obstructive pulmonary disease-smoking comorbidity. *BMC Pulm. Med.* 22:182. 10.1186/s12890-022-01964-6 35524207 PMC9078025

[B101] Zhang Y. Yang Y. Xu X. Yuan Y. (2022). Coupling of spatial and directional functional network connectivity reveals a physiological basis for salience network hubs in asthma. *Brain Imaging Behav.* 16 176–185. 34286477 10.1007/s11682-021-00490-z

[B102] Zhao N. Yuan L. X. Jia X. Z. Zhou X. F. Deng X. P. He H. J. (2018). Intra- and Inter-Scanner Reliability of Voxel-Wise Whole-Brain Analytic Metrics for Resting State fMRI. *Front. Neuroinform.* 12:54. 10.3389/fninf.2018.00054 30186131 PMC6110941

[B103] Zhu L. Zhao J. Yang Y. Shangguan Q. Chen Y. He Y. (2023). Brain network study of attentional cognitive impairment in children with bronchial asthma. *Int. J. Dev. Neurosci.* 83 224–231. 36633998 10.1002/jdn.10250

